# hsa_circ_0013401 Accelerates the Growth and Metastasis and Prevents Apoptosis and Autophagy of Neuroblastoma Cells by Sponging miR-195 to Release PAK2

**DOI:** 10.1155/2021/9936154

**Published:** 2021-11-22

**Authors:** Shibo Zhu, Xiangliang Tang, Xiaofeng Gao, Jingqi Zhang, Yanhong Cui, Dian Li, Wei Jia

**Affiliations:** Department of Pediatric Urology, Guangzhou Women and Children's Medical Center, Guangzhou Medical University, Guangzhou, 510623 Guangdong, China

## Abstract

**Background:**

Increased levels of circRNAs have been identified in a variety of cancers. However, the specific functions and mechanisms of circRNAs in neuroblastoma (NB) have not been fully explored.

**Methods:**

The levels of hsa_circ_0045997, hsa_circ_0080307, hsa_circ_0013401, hsa_circ_0077578, and microRNA-195 were confirmed by RT-qPCR in NB. Gain- and loss-of-function assays and rescue experiments were conducted to determine the influence of hsa_circ_0013401, miR-195, and P21-activated kinase 2 (PAK2) on the proliferation, apoptosis, autophagy, migration, and invasion of NB cells. Regulatory gene targets were validated by the luciferase assay. A xenograft mouse model was used to determine the *in vivo* effects of hsa_circ_0013401.

**Results:**

hsa_circ_0013401 was highly expressed, miR-195 was lowly expressed, and there was a negative correlation between hsa_circ_0013401 and miR-195 in NB. The inhibitory effects of hsa_circ_0013401 knockdown suppressed the proliferation, migration, and invasion and induced the apoptosis and autophagy of NB cells by targeting miR-195 to downregulate PAK2 expression. Luciferase reporter assays showed that miR-195 was a direct target of hsa_circ_0013401, and *PAK2* was the downstream target gene of miR-195. *In vivo* studies showed that hsa_circ_0013401 promotes tumor formation.

**Conclusions:**

hsa_circ_0013401 induced NB progression through miR-195 to enhance PAK2. Therefore, we might highlight a novel regulatory axis (hsa_circ_0013401/miR-195/PAK2) in NB.

## 1. Introduction

Neuroblastoma (NB) is a malignant tumor commonly discovered in the peripheral nervous system of infants [1]. NB is also a common embryonal solid tumor originating from the neural crests of the sympathetic nervous system [2]. Statistics show that the incidence of NB is second only to those of leukemia and brain cancer, and the mortality rate of children with NB is ~15% [3]. Most NB patients are diagnosed with middle or late-stage disease due to the high degree of malignancy, rapid development, and easy metastasis of NB [4]. The current methods for treating NB are surgery, radiotherapy, chemotherapy, stem cell transplantation to enhance the effects of chemotherapy, and an induction of cell differentiation [5]. Despite combination therapy, the survival rate of patients with advanced NB remains low [6]. Therefore, there is an urgent need to identify specific molecules that can be exploited in targeted therapy regimens for NB patients.

Circular RNAs (circRNAs) comprise a class of noncoding RNA molecules without a cap structure at the 5′ ends and have a poly(A) tail at the 3′ ends, which can form a circular structure with covalent bonds [7]. Due to their closed ring structure, circRNAs are stable in cells and not easily degraded by exonuclease [8]. Moreover, circRNAs have developmental stage specificity and tissue specificity and are abundant in cells and highly conserved in different species [9]. Recent studies have shown that circRNAs have significant effects in regulating gene expression [10]. Several studies have demonstrated that circRNAs play critical roles in a variety of cancers, such as circHIPK3 in colorectal cancer [11], circRNA_102171 in thyroid cancer [12], circSLC8A1 in bladder cancer [13], and circCDR1 in gastric cancer [14]. Therefore, circRNAs can serve as diagnostic markers and therapeutic targets for tumors [15]. However, the role played by circRNA in NB development has been rarely investigated, and the biological functions and potential mechanisms of most circRNAs have yet to be discovered.

MicroRNAs (miRNAs), as crucial posttranscriptional regulators, can directly bind to the nontranslational sites of mRNAs via complementary base pairing and thus suppress protein translation or induce mRNA degradation [16, 17]. Recent studies have shown that circRNAs are rich in miRNA reaction elements (MREs) and can reduce the inhibitory effects of miRNAs on target gene expression by sponging miRNAs [7, 18]. Compared with other types of competitive endogenous RNA (ceRNAs), circRNAs contain more binding sites for miRNA and therefore compete with endogenous RNA (ceRNA) [18]. Previous studies have shown that microRNA-195 (miR-195) is expressed at low levels in cancers [19–21], indicating that a low level of miR-195 expression may be related to cancer progression. Besides, recent research also suggested that miR-195 is lowly expressed in NB and miR-195 also can prevent the pyroptosis of NB cells [22]. However, the related circRNAs that can regulate miR-195 expression have been poorly reported in NB. We performed a bioinformatics analysis that revealed the existence of interaction sites between various circRNAs (has_circ_0045997, hsa_circ_0080307, has_circ_0013401, and hsa_circ_0077578) and miR-195. Therefore, we further investigated whether the potential functions and mechanisms of those circRNAs and miR-195 might significantly affect NB treatment.

In our study, we examined the expression levels of 4 circRNAs in NB tissues and discovered that hsa_circ_0013401 was highly expressed in NB. We then verified the targeted regulatory relationship between hsa_circ_0013401 and miR-195 and investigated the effects of hsa_circ_0013401 and miR-195 on the proliferation, apoptosis, autophagy, migration, and invasion of NB cells. Moreover, we confirmed that P21-activated kinase 2 (*PAK2*) was a target gene of miR-195 and proved the crucial roles played by the hsa_circ_0013401/miR-195/PAK2 axis in NB development and progression.

## 2. Methods

### 2.1. Tissue Samples

Samples of gangliocytoma tissue (GN, *n* = 8) and NB tissue (*n* = 8) were collected from patients who were diagnosed at the Guangzhou Women and Children's Medical Center of Guangzhou Medical University (Guangzhou, China). All histopathology assessments were performed by two professional pathologists using a double-blind method. We also provided some basic information regarding the 8 GN and 8 NB patients (Table 1). All clinical studies were conducted in accordance with the Declaration of Helsinki, and each patient provided their written informed consent for study participation. The study protocol was approved by the Ethics Committee of Guangzhou Women and Children's Medical Center of Guangzhou Medical University (Guangzhou, China).

### 2.2. Cell Lines

Six NB cell lines (SK-N-BE, GNP, SH-SY5Y, IMR-32, LAN-1, and SK-N-SH) were provided by the Type Culture Collection of the Chinese Academy of Sciences (Shanghai, China). The cells were cultured in RPMI-1640 medium (HyClone Laboratories Inc., Logan, Utah, USA) containing 10% fetal bovine serum (FBS, Life Technologies, cat. no. 10270) and 1% penicillin/streptomycin (Gibco, Gaithersburg, MD, USA) at 37°C in an incubator with 5% CO_2_.

### 2.3. Fluorescence In Situ Hybridization (FISH)

The samples were added to Carnoy's solution and then centrifuged at 1000 g for 6 minutes. Following centrifugation, 100 *μ*L aliquots of solution were placed onto clean glass slides. Prehybridization was performed by treatment with a hybridization solution (WAKO, cat. no. #544-01331) for 1 hour (h) at 37°C; after which, probes (100 nM) were added to the slides. After denaturation at 74°C for 6 min, the slides were incubated overnight in a humid chamber at 35°C and then washed again with 0.4× SSC at 45°C. The slides were then treated with 2× SSC containing 0.05% Tween-20 at room temperature (RT) for 1 min. After nuclear staining with DAPI (Life Science), the results were visualized with a fluorescence microscope.

### 2.4. Immunohistochemistry (IHC) Assay

GN and NB tissues were soaked in 10% paraformaldehyde (Sigma, St. Louis, MO, USA) at 4°C for 12 h and then dehydrated. After embedding, a rotary microtome (Leica, GER) was used to cut the tissue samples into 3.5 *μ*m thick sections. The sections were immersed in a 42.5°C water bath and then mounted onto microscope slides (Citoglas, China). After deparaffinization and rehydration, the tissue sections were processed using reagents in an SABC kit (Bosterbio, China) according to instructions provided by the manufacturer. After processing, the mounted sections were incubated with anti-Ki67 (1 : 200, Abcam, UK) and anti-PAK2 (1 : 100, Abcam, UK) antibodies at 4°C for 12 h and then stained with reagents in a DAB kit (Bosterbio, China). The staining results were obtained using an inverted microscope (Nikon Eclipse TI-SR, Japan).

### 2.5. RNA Transfection

miR-195 mimics, miR-195 inhibitor, and a negative control (NC) were all designed and obtained from GenePharma (China). circ_0013401-overexpression plasmids, circ_0013401 shRNA, and PAK2-overexpression plasmids were obtained from BioVector (Newark, CA, USA). All cell transfections were performed using Lipofectamine 3000 (Invitrogen, Carlsbad, CA, USA) according to the manufacturer's instructions.

### 2.6. Quantitative Real-Time PCR (RT-PCR)

The total RNA was extracted from NB tissue samples and treated NB cells using the TRIzol reagent (Invitrogen). After inspection, the RNA extracts were reverse transcribed to cDNA using a Bestar™ qPCR RT kit (DBI Bioscience, cat. no. #2220). RT-PCR was performed using Bestar™ qPCR MasterMix (DBI Bioscience, cat. no. #2043). The sequences of the primers used in this study are shown in Table 2. The levels of gene expression were calculated using the 2^−ΔΔCt^ method.

### 2.7. Cell Counting Kit-8 (CCK-8) Assay

The CCK-8 assay (Dojindo, Rockville, MD, USA) was used to evaluate the viability of treated NB cells. In brief, the treated NB cells were seeded into 96-well plates (2 × 10^4^ cells/well) and cultured at 37°C for 24 h. Next, 10 *μ*L of CCK-8 solution was added to each well, and these plates were incubated at 37°C for 10 min. The optical density (OD) of each well was determined at 450 nm.

### 2.8. EdU Assay

Treated SH-SY5Y and SK-N-BE cells were fixed with 0.5% paraformaldehyde (Sigma) and then treated with EdU solution (RioBio, Guangzhou, China) for 30 min. Subsequently, a flow cytometer (Becton Dickinson, Franklin Lakes, NJ, USA) was used to assess cell proliferation.

### 2.9. Colony Formation Assay

Briefly, the SH-SY5Y and SK-N-BE cells in each group were plated into 6-well plates (2000 cells/well) and cultured at 37°C for 14 days. The cells were then fixed with 4% paraformaldehyde and stained with Giemsa solution. The numbers of cell colonies were counted under a microscope.

### 2.10. Transwell Assay

The invasion or migration abilities of SH-SY5Y and SK-N-BE cells in each group were assessed using Transwell chambers (8 *μ*m, Corning, Corning, NY, USA) with or without a Matrigel coating, respectively. Cells (1 × 10^6^ cells) suspended in 500 *μ*L of serum-free medium were seeded into the upper chamber, and 500 *μ*L of culture medium containing 15% FBS was added to the lower chamber. After 24 h of incubation, the invaded or migrated cells were fixed with 4% paraformaldehyde (Sigma) and stained with 5% crystal violet solution (Sigma). The numbers of invaded or migrated cells were counted under a microscope.

### 2.11. Flow Cytometric Analysis

The apoptosis rates of SH-SY5Y and SK-N-BE cells in each group were monitored by flow cytometry performed using the Annexin V/FITC double staining method (BD Biosciences) according to instructions.

### 2.12. Transmission Electron Microscopy (TEM)

After washing, the treated SH-SY5Y and SK-N-BE cells were fixed in 2% glutaraldehyde for 2 h, treated with 1% osmium tetroxide for 1 h, and then dehydrated using a graded ethanol series for 2 h. TEM observations were carried out using a Leo 912 AB electron microscope.

### 2.13. Immunofluorescence Staining

After fixation, the treated SH-SY5Y and SK-N-BE cells were incubated with 5% Tween-20 for 2 h. The cells were then blocked with 10% normal goat serum for 1 h and subsequently incubated overnight with anti-LC3 (1 : 100, Abcam, cat. no. ab62720) and anti-PAK2 (Abcam, cat. no. ab3442) primary antibodies, followed by incubation with a secondary antibody (Abcam, cat. no. DAR-546) for 2 h. The cell nucleus was stained with DAPI (Life Science) for 30 min, and results were photographed using a confocal laser microscope (Zeiss, Germany, LSM710).

### 2.14. Western Blotting

The total proteins were extracted using RIPA buffer (Beyotime, Shanghai, China), and the protein concentration in each extract was detected using a BCA kit (Beyotime, China). A 50 *μ*g sample of protein from each extract was separated by SDS-PAGE (10%), and the protein bands were transferred onto PVDF membranes (Millipore, Burlington, MA, USA), which were subsequently blocked with 5% nonfat milk for 2 h. Next, the membranes were incubated overnight with primary antibodies, followed by incubation with an HRP-conjugated secondary antibody for 1.5 h. Signals were detected by using enhanced chemiluminescence (ECL, Thermo Fisher Scientific, Waltham, MA, USA) and analyzed using ImageJ software.

### 2.15. In Vivo Tumor Growth Assay

Male BALB/c nude mice (8 weeks old) were provided by the Laboratory Animal Center of Southern Medical University (no. 44002100023925). The protocol for the *in vivo* study was approved by the Institutional Animal Ethics Committee of Guangzhou Medical University (Guangzhou, China), and the study was carried out at the laboratory animal center. Briefly, transfected SH-SY5Y cells (200 *μ*L; 1 × 10^7^ cells) were subcutaneously injected into the left flank of nude mice; after which, tumor size was measured every 7 days up to 28 days after injection. After 28 days, the mice were sacrificed by anesthesia with sodium pentobarbital and the xenograft tumors were excised. The tumor volumes were calculated using the formula length × width^2^ × 0.5.

### 2.16. Dual-Luciferase Reporter Assay

To verify the relationship between circ_0013401 and miR-195 in SH-SY5Y and SK-N-BE cells, circ_0013401-wild type (WT) and circ_0013401-mutant (Mut) plasmids were constructed using the WT and Mut fragments of circ_0013401, including the putative miR-195 binding sites and psiCheck-2 vector (Promega, cat. no. C8021). Next, SH-SY5Y or SK-N-BE cells were added to the wells of 6-well plates (1 × 10^4^ cells/well) and incubated overnight at 37°C. The cells were then cotransfected with miR-195 mimics plus the circ_0013401-WT or circ_0013401-Mut. The firefly and Renilla luciferase activities of the SH-SY5Y and SK-N-BE cells in each group were examined using a Dual-Luciferase Assay System (Promega, Madison, WI, USA).

### 2.17. Statistical Analysis

All data were analyzed using IBM SPSS Statistics for Windows, version 20.0 software (IBM Corp., Armonk, NY, USA). Results are expressed as the mean value ± SEM of data obtained from three independent experiments. The significance of differences between groups was analyzed by Student's *t*-test or one-way analysis of variance. A *p* value < 0.05 was considered to be statistically significant.

## 3. Results

### 3.1. circ_0013401 Was Highly Expressed in NB

**Figure 1:**
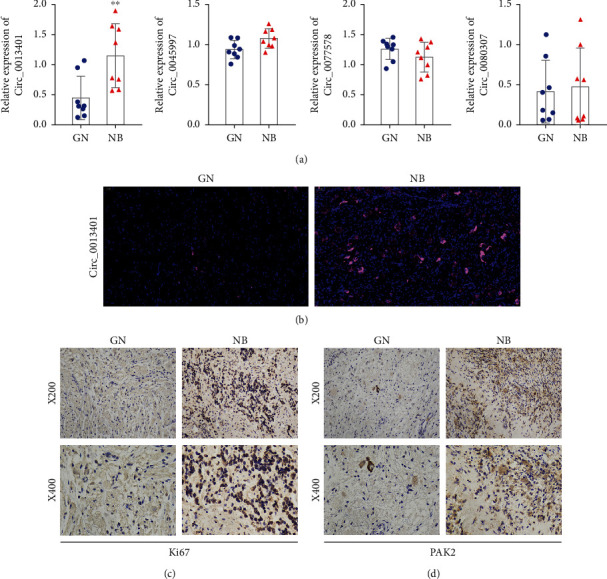
circ_0013401 was highly expressed in NB. (a) The levels of circ_0013401, circ_0045997, circ_0077578, and circ_0080307 expression in samples of GN and NB tissues were confirmed (*n* = 8 tissues per group). (b) circ_0013401 expression in GN and NB tissues was examined by FISH. Magnification, ×200. IHC assays were performed to examine the expression of Ki67 (c) and PAK2 (d) in GN and NB tissues. Magnification, ×200; magnification, ×400. ^∗∗^*p* < 0.01.

To investigate the changes that occurred in circRNA expression in NB, we first examined the levels of circ_0013401, circ_0045997, circ_0077578, and circ_0080307 expression in 8 gangliocytoma (GN) and 8 neuroblastoma (NB) tissue samples. As shown in Figure 1(a), only circ_0013401 expression was significantly upregulated in the NB tissues when compared with the GN tissues (*p* < 0.01). Next, we performed a FISH assay to identify circ_0013401 expression in GN and NB tissues and found that circ_0013401 was much more highly expressed in the NB tissues than in the GN tissues (Figure 1(b)). Additionally, the levels of Ki67 and PAK2 expression were examined using IHC assays, and those results showed that expression of both Ki67 and PAK2 was markedly elevated in the NB tissues when compared with the GN tissues (Figures 1(c) and 1(d)). Consequently, we confirmed that circ_0013401, Ki67, and PAK2 were highly expressed in NB.

### 3.2. circ_0013401, as an Oncogene, Significantly Accelerated NB Cell Proliferation

**Figure 2:**
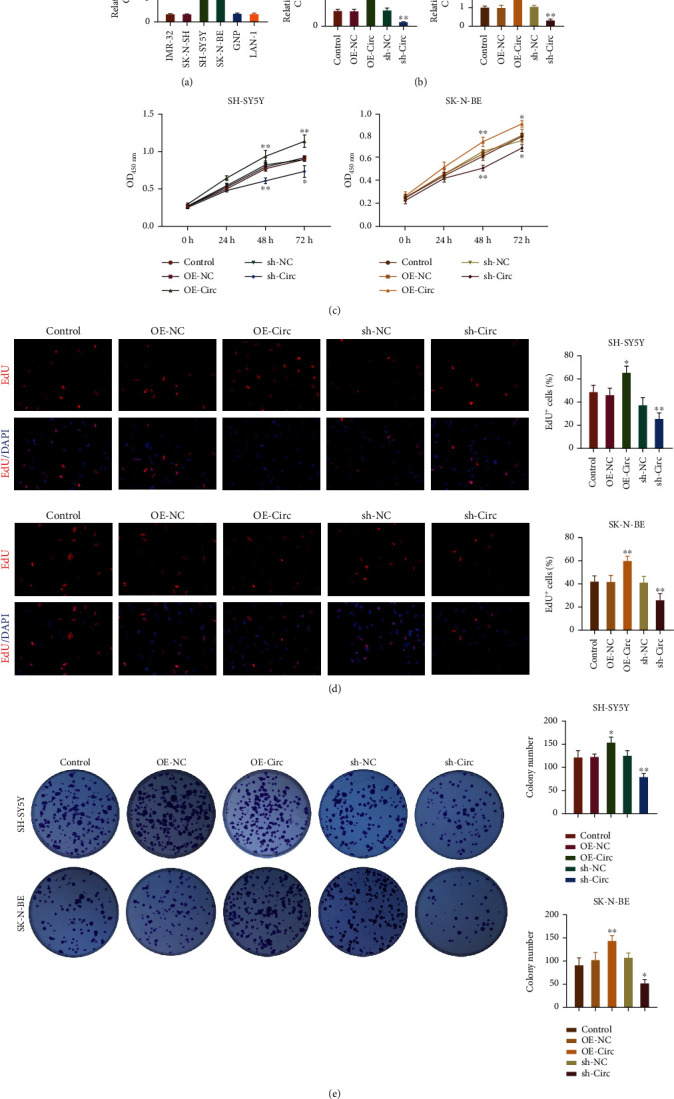
circ_0013401, as an oncogene, significantly increased NB cell proliferation. (a) circ_0013401 expression in different neuroblastoma cell lines (SK-N-BE, GNP, SH-SY5Y, IMR-32, LAN-1, and SK-N-SH) was determined by RT-qPCR. (b) SH-SY5Y and SK-N-BE cells were transfected with a circ_0013401-overexpression plasmid or circ_0013401 shRNA, and the resultant effects were examined using RT-qPCR assays. (c) CCK-8 assays were performed to confirm the effect of circ_0013401 overexpression or knockdown on the proliferation of SH-SY5Y and SK-N-BE cells. Cell proliferation was also assessed by EdU staining (d) and the clone formation assay (e) in SH-SY5Y and SK-N-BE cells with circ_0013401 overexpression or silencing. ^∗^*p* < 0.05, ^∗∗^*p* < 0.01.

Given that circ_0013401 was upregulated in NB tissues, we investigated the effects of circ_0013401 overexpression and knockdown on various functions in NB cells. We first examined the levels of circ_0013401 expression in different neuroblastoma cell lines (SK-N-BE, GNP, SH-SY5Y, IMR-32, LAN-1, and SK-N-SH) using RT-qPCR and found that circ_0013401 expression was significantly upregulated in SH-SY5Y and SK-N-BE cells when compared with other NB cell lines. Therefore, SH-SY5Y and SK-N-BE cells were selected for use in subsequent experiments (*p* < 0.01, Figure 2(a)). RT-qPCR analyses were performed to determine how transfection with a circ_0013401-overexpression plasmid or circ_0013401 shRNA might affect SH-SY5Y and SK-N-BE cells. As shown in Figure 2(b), circ_0013401 expression was upregulated in the circ_0013401 overexpression group when compared with the overexpression-NC group. Furthermore, circ_0013401 expression was significantly downregulated in the circ_0013401 knockdown group when compared with the sh-NC group (*p* < 0.01, Figure 2(b)). Next, we examined how circ_0013401 overexpression or knockdown affected the proliferation of SH-SY5Y and SK-N-BE cells. Results of CCK-8 assays showed that SH-SY5Y and SK-N-BE cells in the circ_0013401 overexpression groups were significantly more viable than those in the overexpression-NC group, while cell viability was dramatically decreased in the circ_0013401 knockdown groups relative to the sh-NC group (*p* < 0.05, *p* < 0.01, Figure 2(c)). Consequently, the numbers of EdU^+^ in circ_0013401-overexpressing SH-SY5Y and SK-N-BE cells were significantly higher than those in the overexpression-NC group, and the numbers of EdU^+^ circ_0013401-silenced cells were dramatically lower than those in the sh-NC group (*p* < 0.05, *p* < 0.01, Figure 2(d)). Meanwhile, clone formation assays revealed that circ_0013401 overexpression induced NB cell proliferation, and circ_0013401 knockdown suppressed NB cell proliferation (*p* < 0.05, *p* < 0.01, Figure 2(e)). These findings verified that circ_0013401 played a significant role in inducing the proliferation of NB cells.

### 3.3. circ_0013401 Facilitated the Migration and Invasion of NB Cells and Prevented Their Apoptosis and Autophagy

**Figure 3:**
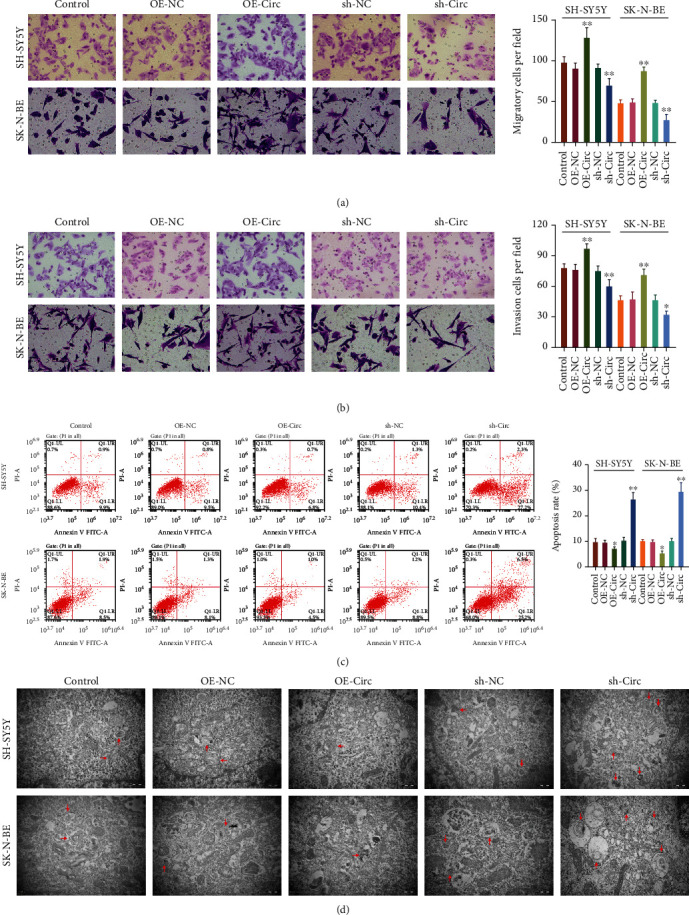
circ_0013401 markedly facilitated NB cell migration and invasion and prevented NB cell apoptosis and autophagy. Transwell assays were performed to identify the effects of circ_0013401 overexpression or knockdown on the migration (a) and invasion (b) capabilities of SH-SY5Y and SK-N-BE cells; the numbers of migrated and invading cells were counted. (c) The effects of circ_0013401 overexpression or knockdown on the apoptosis of SH-SY5Y and SK-N-BE cells were evaluated by flow cytometry, and the numbers of apoptotic cells in each group were calculated. (d) Changes in autophagy (red arrow) that occurred in SH-SY5Y and SK-N-BE cells with circ_0013401 overexpression or knockdown were examined by TEM. ^∗^*p* < 0.05, ^∗∗^*p* < 0.01.

We next sought to further verify the changes in migration, invasion, apoptosis, and autophagy that occurred in SH-SY5Y and SK-N-BE cells following circ_0013401 overexpression or knockdown. First, the migration and invasion capabilities of transfected SH-SY5Y and SK-N-BE cells were investigated using Transwell assays. The resultant data showed that overexpression of circ_0013401 enhanced the migration and invasion capabilities of the cells, while circ_0013401 knockdown led to significant reductions in SH-SY5Y and SK-N-BE cell migration and invasion (*p* < 0.05, *p* < 0.01, Figures 3(a) and 3(b)). Data from flow cytometry studies indicated that circ_0013401 overexpression notably reduced the apoptosis rate of both cell lines, and circ_0013401 knockdown significantly increased the apoptosis rates of SH-SY5Y and SK-N-BE cells (*p* < 0.05, *p* < 0.01, Figure 3(c)). Moreover, transmission electron microscopy (TEM) studies revealed that when compared to cells in the control groups, the numbers of autophagosomes in circ_0013401-overexpressing SH-SY5Y and SK-N-BE cells were significantly reduced, while large numbers of autophagosomes were seen in the circ_0013401-silenced SH-SY5Y and SK-N-BE cells (Figure 3(d)). These findings indicated that circ_0013401 could induce migration and invasion and repress apoptosis and autophagy in SH-SY5Y and SK-N-BE cells.

### 3.4. circ_0013401 Regulated the miR-195/PAK2 Axis, as well as Autophagy- and Apoptosis-Related Proteins in NB Cells

**Figure 4:**
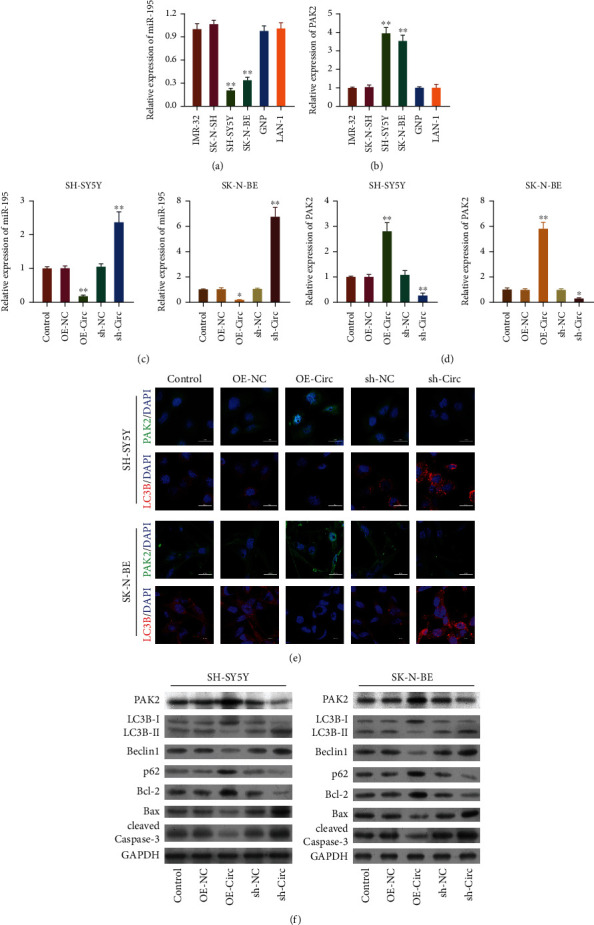
circ_0013401 regulated miR-195, PAK2, and autophagy- and apoptosis-related proteins in NB cells. (a) RT-qPCR analysis of miR-195 expression in different NB cell lines (SK-N-BE, GNP, SH-SY5Y, IMR-32, LAN-1, and SK-N-SH). (b) PAK2 expression was tested using RT-qPCR in different neuroblastoma cell lines (SK-N-BE, GNP, SH-SY5Y, IMR-32, LAN-1, and SK-N-SH). (c) The effects of circ_0013401 overexpression or knockdown on miR-195 expression were assessed by RT-qPCR. (d) The changes in PAK2 expression that occurred in SH-SY5Y and SK-N-BE cells with circ_0013401 overexpression or silencing were evaluated by RT-qPCR. (e) PAK2 and LC3B expression in SH-SY5Y and SK-N-BE cells with circ_0013401 overexpression or knockdown was determined using IF assays. Magnification, ×400; scale bar = 20 *μ*m. (f) Western blotting was used to examine the levels of PAK2, LC3B, Beclin1, p62, Bcl-2, Bax, and cleaved caspase-3 in SH-SY5Y and SK-N-BE cells with circ_0013401 overexpression or silencing. ^∗^*p* < 0.05, ^∗∗^*p* < 0.01.

Subsequently, we sought to identify the biological pathway that regulates circ_0013401 in NB. Bioinformatics predictions indicated that circ_0013401 might be a miRNA response element (MRE) of miR-195, which might bind with circ_0013401. Those predications also suggested *PAK2* as the most likely target gene of miR-195. Therefore, miR-195 and PAK2 became the focus of our mechanistic studies. To further confirm the levels of miR-195 expression in NB, the levels of miR-195 expression in different neuroblastoma cell lines were analyzed using the RT-qPCR. As indicated in Figure 4(a), miR-195 expression was significantly downregulated in SH-SY5Y and SK-N-BE cells when compared with the other cell lines (*p* < 0.01). Meanwhile, we discovered that PAK2 expression was dramatically elevated in SH-SY5Y and SK-N-BE cells relative to the other cell lines (*p* < 0.01, Figure 4(b)). In addition, we demonstrated that miR-195 could be downregulated by circ_0013401 overexpression and upregulated by circ_0013401 knockdown in SH-SY5Y and SK-N-BE cells (*p* < 0.05, *p* < 0.01, Figure 4(c)). In contrast, PAK2 could be markedly upregulated by circ_0013401 overexpression and dramatically downregulated by circ_0013401 knockdown in SH-SY5Y and SK-N-BE cells (*p* < 0.05, *p* < 0.01, Figure 4(d)). Meanwhile, results of IF studies indicated that circ_0013401 overexpression increased PAK2 expression and reduced LC3B expression; meanwhile, circ_0013401 knockdown significantly decreased PAK2 expression and increased LC3B expression in SH-SY5Y and SK-N-BE cells (Figure 4(e)). More importantly, western blot studies showed that circ_0013401 overexpression could upregulate the levels of PAK2, p62, and Bcl-2 and downregulate the levels of LC3B II/I, Beclin1, Bax, and cleaved caspase-3 in SH-SY5Y and SK-N-BE cells, while circ_0013401 knockdown produced the opposite effects on these proteins in SH-SY5Y and SK-N-BE cells (Figure 4(f)). When taken together, these findings indicated that circ_0013401 dramatically suppressed autophagy and apoptosis and regulated the miR-195/PAK2 axis in NB cells.

### 3.5. circ_0013401 Sponged miR-195, and PAK2 Was a Target Gene of miR-195

**Figure 5:**
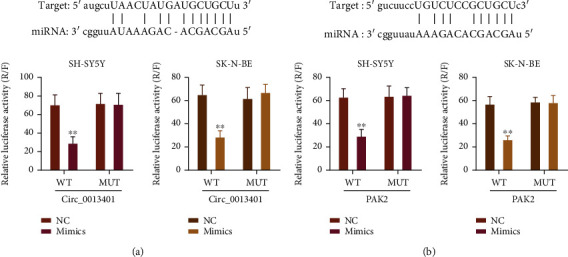
circ_0013401 sponged miR-195, and *PAK2* was a target gene of miR-195. (a) Dual-luciferase reporter assays were conducted to investigate the interplay between circ_0013401 and miR-195. (b) A dual-luciferase reporter assay proved that *PAK2* was a direct target gene of miR-195.

Luciferase reporter assays were performed to verify the relationship between miR-195 and circ_0013401 or PAK2. Our results showed that miR-195 could significantly reduce the luciferase activity of WT-circ_0013401, but not of Mut-circ_0013401 in SH-SY5Y and SK-N-BE cells (*p* < 0.01, Figure 5(a)). Similarly, the luciferase activity of WT-PAK2 was significantly reduced by miR-195, while the luciferase activity of Mut-PAK2 was not affected by miR-195 in SH-SY5Y and SK-N-BE cells (*p* < 0.01, Figure 5(b)). Thus, we proved that circ_0013401 could regulate the miR-195/PAK2 axis via targeted binding.

### 3.6. The Inhibitory Effect of circ_0013401 Knockdown on the Proliferation of NB Cells Could Be Reversed by an miR-195 Inhibitor

**Figure 6:**
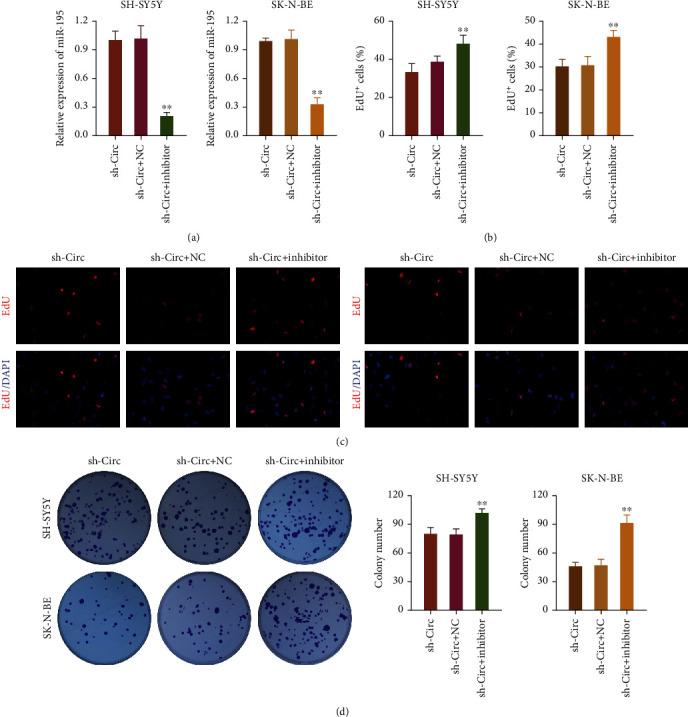
An miR-195 inhibitor reversed the inhibitory effect of circ_0013401 knockdown on NB cell proliferation. (a) circ_0013401-silenced SH-SY5Y and SK-N-BE cells were transfected with an miR-195 inhibitor or NC, and miR-195 expression was confirmed by RT-qPCR. (b, c) SH-SY5Y and SK-N-BE cells were cotransfected with circ_0013401 shRNA and the miR-195 inhibitor, and cell proliferation was examined by EdU staining. The percentage of EdU^+^ cells was calculated. (d) Clone formation assays were also performed to assess the proliferation of cotransfected SH-SY5Y and SK-N-BE cells. ^∗∗^*p* < 0.01.

Next, rescue assays were performed to confirm whether circ_0013401 induced NC proliferation by targeting miR-195. An miR-195 inhibitor was transfected into circ_0013401-silenced SH-SY5Y and SK-N-BE cells, and a subsequent RT-qPCR analysis showed that the miR-195 inhibitor markedly downregulated miR-195 expression, which was induced by circ_0013401 shRNA in SH-SY5Y and SK-N-BE cells (*p* < 0.01, Figure 6(a)). EdU staining results revealed that the proliferation of SH-SY5Y and SK-N-BE cells was significantly increased in the cotransfection groups (miR-195 inhibitor plus circ_0013401 shRNA) when compared with proliferation in the circ_0013401 shRNA transfection groups (*p* < 0.01, Figures 6(b) and 6(c)). Likewise, results of clone formation assays revealed that the miR-195 inhibitor facilitated NB cell proliferation mediated by circ_0013401 knockdown (*p* < 0.01, Figure 6(d)). These findings proved that the inhibition of cell proliferation mediated by circ_0013401 shRNA in NB could be significantly reversed by an miR-195 inhibitor.

### 3.7. miR-195 Was Involved in the Inhibition of Migration and Invasion and Induction of Apoptosis Mediated by circ_0013401 Knockdown in NB Cells

**Figure 7:**
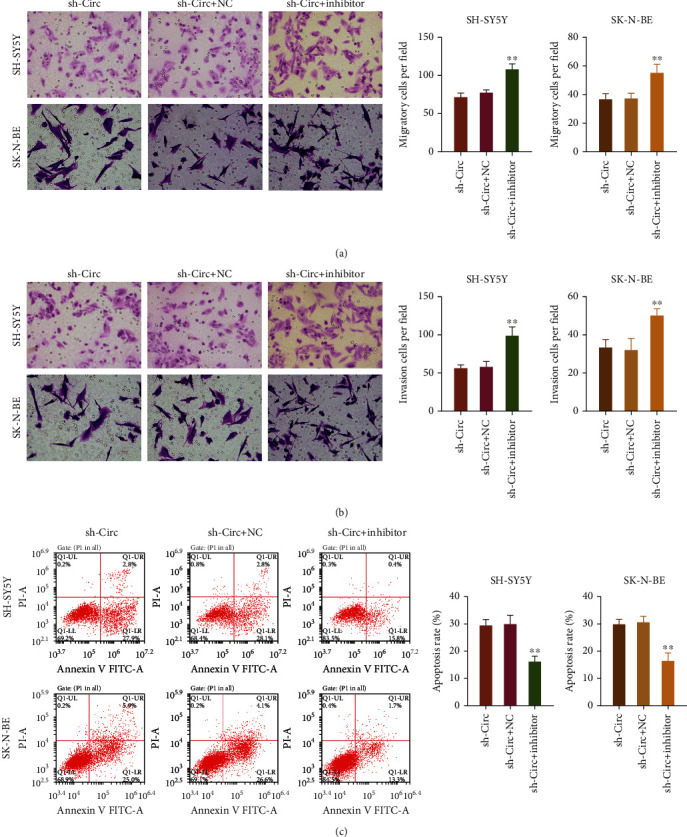
miR-195 was involved in the inhibition of migration and invasion and induction of apoptosis mediated by circ_0013401 knockdown in NB cells. (a, b) The effects of circ_0013401 shRNA and the miR-195 inhibitor on the migration and invasion of SH-SY5Y and SK-N-BE cells were examined by Transwell assays. Magnification, ×200. (c) The apoptosis rates of SH-SY5Y and SK-N-BE cells cotransfected with circ_0013401 shRNA and the miR-195 inhibitor were analyzed by flow cytometry. ^∗∗^*p* < 0.01.

Transwell assays revealed that the migration and invasion abilities of SH-SY5Y and SK-N-BE cells in the cotransfection groups (miR-195 inhibitor plus circ_0013401 shRNA) were markedly higher than those in the circ_0013401 shRNA transfection groups (*p* < 0.01, Figures 7(a) and 7(b)). Flow cytometry results showed that transfection with the miR-195 inhibitor significantly reduced the apoptosis rates of SH-SY5Y and SK-N-BE cells, which had been induced by miR-491-5p mimics (*p* < 0.01, Figure 7(c)). In general, our findings verified that circ_0013401 knockdown prevented the migration and invasion and accelerated the apoptosis of NB cells by sponging miR-195.

### 3.8. The miR-195 Inhibitor Attenuated the Downregulation Effects of circ_0013401 Knockdown on PAK2 and Autophagy- and Apoptosis-Related Proteins in NB Cells

**Figure 8:**
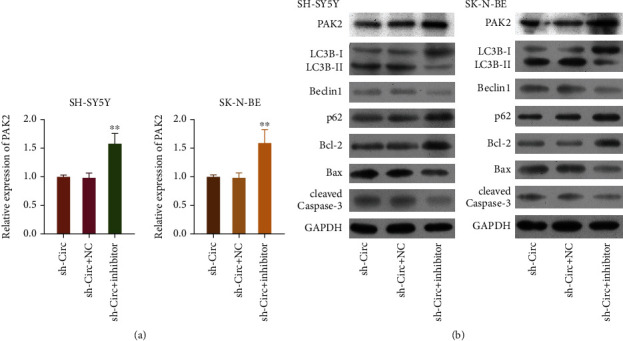
Treatment with an miR-195 inhibitor attenuated the downregulation effects of circ_0013401 knockdown on PAK2 and autophagy- and apoptosis-related proteins in NB cells. (a) The levels of PAK2 in circ_0013401 shRNA and miR-195 inhibitor-transfected SH-SY5Y and SK-N-BE cells were examined by RT-qPCR. (b) The levels of PAK2, LC3B, Beclin1, p62, Bcl-2, Bax, and cleaved caspase-3 protein expression in SH-SY5Y and SK-N-BE cells cotransfected with circ_0013401 shRNA and the miR-195 inhibitor were examined by western blotting. ^∗∗^*p* < 0.01.

We next sought to identify downstream molecules that regulated the circ_0013401/miR-195 axis in NB cells. SH-SY5Y and SK-N-BE cells were cotransfected with circ_0013401 shRNA plus the miR-195 inhibitor; after which, RT-qPCR analyses were performed to determine the levels of PAK2 expression. Those data indicated that PAK2 expression mediated by circ_0013401 knockdown in SH-SY5Y and SK-N-BE cells was dramatically upregulated by the miR-195 inhibitor (*p* < 0.01, Figure 8(a)). Western blot results showed that after transfection with the miR-195 inhibitor, the levels of PAK2, p62, and Bcl-2 expression were upregulated, while the levels of LC3BII/I, Beclin1, Bax, and cleaved caspase-3 expression were downregulated in the circ_0013401 shRNA-transfected SH-SY5Y and SK-N-BE cells (Figure 8(b)). These results suggested that in NB cells, circ_0013401 knockdown could reduce PAK2 expression and induce autophagy and the synthesis of apoptosis-related proteins by altering the levels of miR-195.

### 3.9. Overexpression of PAK2 Suppressed the Apoptosis and Autophagy Mediated by miR-195 in NB Cells

**Figure 9:**
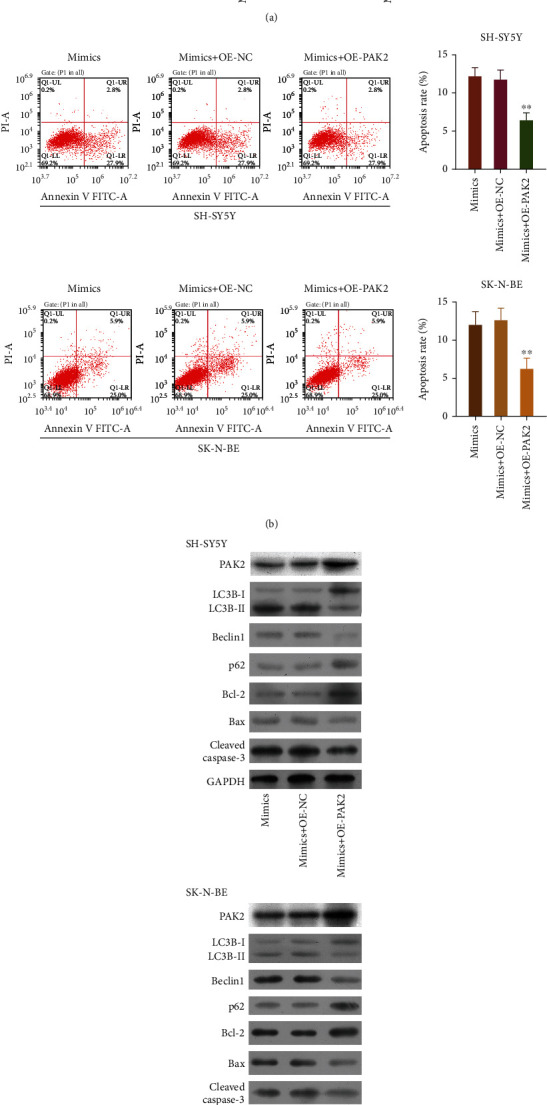
Overexpression of PAK2 suppressed the apoptosis and autophagy mediated by miR-195 in NB cells. SH-SY5Y and SK-N-BE cells were transfected with miR-195 mimics and/or PAK2-overexpression plasmids, respectively. (a) PAK2 expression was assessed by RT-qPCR. (b) Cell apoptosis was assessed by flow cytometry, and the apoptosis rates were calculated. (c) Western blot results for PAK2, LC3B, Beclin1, p62, Bcl-2, Bax, and cleaved caspase-3 proteins. ^∗∗^*p* < 0.01.

Rescue assays were performed to further verify the effects of the miR-195/PAK2 axis on apoptosis and autophagy in NB cells. miR-195 mimics and/or PAK2-overexpression plasmids were transfected into SH-SY5Y and SK-N-BE cells, and PAK2 expression was assessed using the RT-qPCR. As shown in Figure 9(a), overexpression of PAK2 significantly increased PAK2 expression in the miR-195 mimic-transfected SH-SY5Y and SK-N-BE cells (*p* < 0.01). Flow cytometry data revealed that the apoptosis rates of those transfected cells were markedly decreased by PAK2 overexpression (*p* < 0.01, Figure 9(b)). These findings indicated that overexpression of PAK2 also could significantly increase p62 and Bcl-2 expression and decrease the levels of LC3BII/I, Beclin1, Bax, and cleaved caspase-3 expression in miR-195 mimic-transfected SH-SY5Y and SK-N-BE cells (Figure 9(c)). Overall, the studies showed that PAK2 was significantly involved in the effects of miR-195 on apoptosis and autophagy in NB cells.

### 3.10. Verification of the circ_0013401/miR-195/PAK2 Axis in an In Vivo Experiment

**Figure 10:**
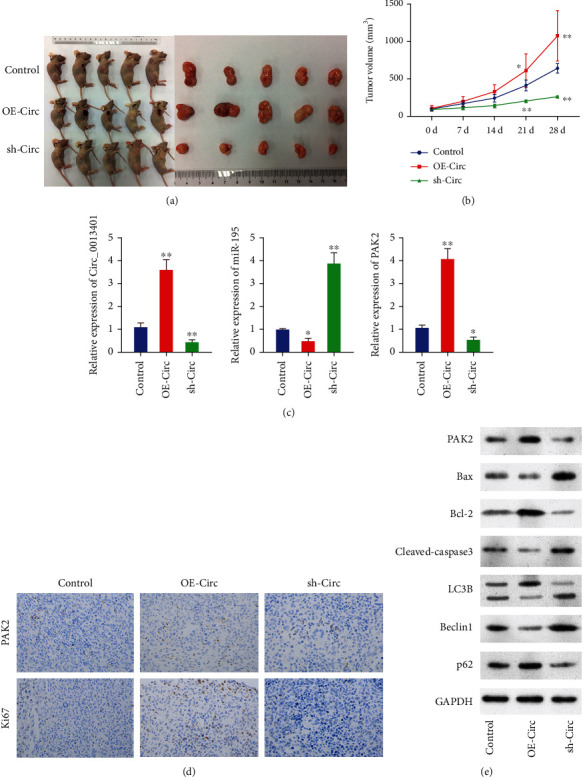
*In vivo* verification of the circ_0013401/miR-195/PAK2 axis. (a) Nude mice were injected with circ_0013401-overexpressing or circ_0013401-silenced SH-SY5Y cells, and representative images of tumors are shown. (b) Tumor volumes were calculated on days 0, 7, 14, 21, and 28. (c) After circ_0013401 overexpression or knockdown, the levels of circ_0013401, miR-195, and PAK2 expression were examined by RT-qPCR. (d) IHC assays were performed to detect PAK2 and Ki67 expression. (e) The levels of apoptosis- and autophagy-related protein expression were examined by western blotting. ^∗^*p* < 0.05, ^∗∗^*p* < 0.01.

Based on results of our *in vitro* experiments, we sought to further verify the regulatory effects of circ_0013401 on NB tumor growth and miR-195/PAK2 expression *in vivo*. We first established cultures of transfected SH-SY5Y cells that either overexpressed circ_0013401 or were silenced and then injected the transfected cells into groups of BALB/c nude mice. After 28 days, the mice were sacrificed and the tumors were removed. We found that NB tumor growth was markedly promoted by circ_0013401 overexpression and suppressed by circ_0013401 knockdown (*p* < 0.01) (Figures 10(a) and 10(b)). Additionally, RT-qPCR results revealed that circ_0013401 and PAK2 expression was significantly upregulated and miR-195 expression was dramatically downregulated in the circ_0013401-overexpression group when compared with the control group. Furthermore, circ_0013401 and PAK2 expression was notably downregulated, and miR-195 expression was significantly upregulated in the circ_0013401-silenced group when compared with the control group (*p* < 0.05, *p* < 0.01, Figure 10(c)). IHC results also showed that circ_0013401 overexpression notably increased the levels of PAK2 and Ki67 expression, and circ_0013401 knockdown dramatically lowered the levels of PAK2 and Ki67 expression in NB tumor tissues (Figure 10(d)). Moreover, we further verified that overexpression of circ_0013401 markedly upregulated the levels of PAK2, p62, and Bcl-2 expression and downregulated LC3B II/I, Beclin1, Bax, and cleaved caspase-3 expression in mouse tumor tissues; knockdown of circ_0013401 produced the opposite effects on the expression of all these proteins in mouse tumor tissues (Figure 10(e)). Hence, we further verified that circ_0013401 could significantly downregulate miR-195 and upregulate PAK2 and inhibit apoptosis and the production of autophagy-related proteins *in vivo*.

## 4. Discussion

NB is the most common extracranial malignant tumor diagnosed in children and accounts for 17% of all childhood cancer-related deaths [23]. For high-risk NB children, the therapeutic effect of comprehensive treatment is very limited, and adverse reactions can be easily observed [24]. Therefore, there is an urgent need to explore the pathogenesis of NB and develop new strategies for treating NB patients. Numerous circRNAs have been reported to play a role in a variety of diseases, especially cancer [25, 26]. Studies have shown that circRNAs can dramatically affect the biological processes of cancers, including cancer cell differentiation, proliferation, apoptosis, migration, invasion, and autophagy [27, 28]. Due to their specificity and stability, circRNAs have joined miRNAs and lncRNAs in becoming a hotspot for clinical research [29]. However, the roles played by some circRNAs in NB remain largely unclear. In the present study, we used RT-qPCR to examine the expression of circ_0013401, circ_0045997, circ_0077578, and circ_0080307 in NB tissues and cells and found that circ_0013401 was dramatically upregulated in NB tissues and cells. Thus, we successfully screened out the potential circRNA (circ_0013401) that can influence NB processes.

Tumor progression is a somatic mutation [30]. Overexpression and silencing of certain genes in tumor cells lead to differences in protein expression from surrounding cells or tissues, and then, tumor cells exhibit different biological behaviors, including excessive proliferation, enhanced migration and invasion capabilities, and apoptosis inhibition [31, 32]. Current studies on NB also focus on inhibiting proliferation, migration, and invasion and inducing apoptosis of tumor cells [33]. In our study, *in vitro* and *in vivo* experiments revealed that hsa_circ_0013401 could induce the proliferation of NB cells and the growth of NB tumors. Moreover, our data also proved that hsa_circ_0013401 could markedly facilitate the migration and invasion of NB cells and prevent their apoptosis. Therefore, we indicated that hsa_circ_0013401 could accelerate NB progression.

Autophagy, as a basic physiological phenomenon, is a key mechanism to maintain cell homeostasis [34]. Abnormal regulation of autophagy is relevant to tumor inflammation, energy metabolism, programmed cell death, and drug resistance [35]. Cells can obtain energy during the autophagic process under various cellular stresses, including nutrient depletion, ischemia, and oxidative stress [36]. In tumor cells, autophagy may act as a self-defensive mechanism which contributes to tumor cell survival by removing toxins and garbage [37], while abnormal autophagic activity may cause the inappropriate degradation of cell components that are indispensable for tumor cell survival, resulting in autophagic cell death [38]. Currently, circRNAs have been certified to have a key regulatory effect on tumor cell autophagy [39, 40]. And our research first verified that circ_0013401 could notably repress NB cell autophagy. However, the role of circ_0013401 in NB cell autophagy is not entirely clear, and further study also is needed to validate the change in autophagic flux in NB cells using inhibitors of autophagosome-lysosome fusion or autophagolysosome hydrolases.

Based on the theory of ceRNAs, circRNAs could be involved in the tumorigenesis of cancers by functioning as miRNA spongers to regulate the expression of their target mRNAs [41, 42]. The specific binding of miRNAs has been considered to be an instinctive function of circRNAs in the circRNA-miRNA-mRNA axis [43]. Therefore, we performed a bioinformatics analysis to construct the hypothetical circRNA-miRNA-mRNA networks of circ_0013401. We also proved that circ_0013401 could act as a sponge for miR-195 and verified that miR-195 directly interacted with circ_0013401 and PAK2 in NB cells. Moreover, we showed that circ_0013401 could indirectly regulate PAK2 expression in NB cells by sponging miR-195. Other recent studies have also suggested that miR-195 is closely related to the progression of numerous cancers, including glioma [44], acute myeloid leukemia [45], bladder cancer [46], breast cancer [47], and cervical cancer [48]. In our study, we further demonstrated that an miR-195 inhibitor could reverse the inhibition of cell proliferation, migration, and invasion and induction of cell apoptosis mediated by circ_0013401 knockdown in NB cells. Moreover, we found that circ_0013401 could upregulate PAK2 expression in NB by targeting miR-195.

PAK belongs to the serine/threonine kinase family of proteins, all of which are highly conserved [49]. As a member of the PAK family, PAK is a downstream effector of GTPase in the Rho family [50]. Multiple studies have proven that PAK2 significantly affects cell proliferation, movement, and apoptosis [51, 52]. Abnormal PAK2 function can result in the occurrence of various diseases (including tumors) [50, 53, 54]. A previous study reported that the combination of Rac, Cdc42, and PAK2 could activate PAK2 to induce cell proliferation and growth [55]. Moreover, PAK2 can be hydrolyzed by aspartic and cysteine peptidases and play a role in regulating cell apoptosis [56]. Current studies have verified that PAK2 is overexpressed in a variety of malignant tumor cells, including lung cancer [57], gastric cancer [58], pancreatic cancer [59], and breast cancer [60]. In our study, we further demonstrated that PAK2 was significantly upregulated in NB and that PAK2 expression could be significantly reduced by miR-195 and elevated by circ_0013401 overexpression in NB. Additionally, we found that overexpression of PAK2 could also dramatically suppress the apoptosis and autophagy mediated by miR-195 in NB cells.

## 5. Conclusions

**Figure 11:**
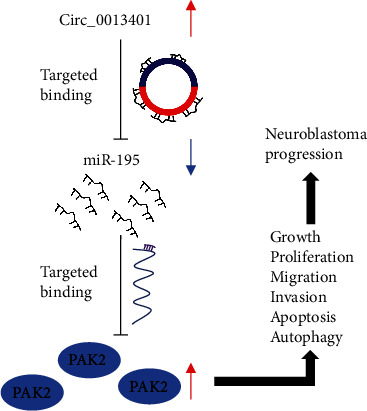
A schematic diagram showing how hsa_circ_0013401 functions in NB. Excess circ_0013401 sponges miR-195 and thereby causes an increase in PAK2 expression, which subsequently promotes the growth, proliferation, migration, and invasion, but prevents the apoptosis and autophagy of NB cells.

Our findings prove that the circ_0013401/miR-195/PAK2 axis plays crucial roles in NB progression both *in vitro* and *in vivo* (Figure 11). Therefore, our results further increase our understanding of NB pathogenesis and can assist in identifying new therapeutic targets for use in treating NB patients.

## Figures and Tables

**Table 1:** Basic information for the 8 GN and 8 NB patients.

Patient ID		Age	Gender	Location	NSE
1	GN	7 years	Female	Adrenal gland	27.98
2	GN	7 years	Female	Abdomen	14.67
3	GN	16 years	Female	Pelvic cavity	13.12
4	GN	5 years	Male	Abdomen	30.38
5	GN	6 years	Male	Abdomen	16.87
6	GN	4 years	Male	Abdomen	23.41
7	GN	10 years	Male	Pelvic cavity	26.43
8	GN	11 years	Male	Adrenal gland	37.28
9	NB	3 years	Male	Adrenal gland	>370
10	NB	1 year	Female	Adrenal gland	50.36
11	NB	3 years	Male	Adrenal gland	42.17
12	NB	2 years	Male	Abdomen	>370
13	NB	5 years	Male	Abdomen	58.69
14	NB	3 months	Female	Adrenal gland	47.5
15	NB	10 months	Female	Adrenal gland	34.12
16	NB	11 months	Female	Abdomen	>370

**Table 2:** Primer sequences used in the RT-qPCR assay.

Name	Sequence (5′-3′)
GAPDH forward	TGTTCGTCATGGGTGTGAAC
GAPDH reverse	ATGGCATGGACTGTGGTCAT
PAK2 forward	CACCCGCAGTAGTGACAGAG
PAK2 reverse	GGGTCAATTACAGACCGTGTG
U6 forward	CTCGCTTCGGCAGCACA
U6 reverse	AACGCTTCACGAATTTGCGT
hsa-miR-195-5p forward	CTCAACTGGTGTCGTGGAGTCGGCAATTCAGTTGAGGCCAATAT
hsa-miR-195-5p reverse	ACACTCCAGCTGGGTAGCAGCACAGAAATAT
hsa_circ_0013401 forward	GTCCTGACTTGTCATGTGCTG
hsa_circ_0013401 reverse	CAGACATTCACAAAGGAGCAA
hsa_circ_0080307 forward	TGCTGCTAAAACCTGTCCAAC
hsa_circ_0080307 reverse	CCACAGCAGCAATACGAACC
hsa_circ_0077578 forward	TGGATGAGATGCCGGTCAA
hsa_circ_0077578 reverse	TAAAGCATGCATCTGTGCGT
hsa_circ_0045997 forward	CTGCGTTTGGAGCCGTT
hsa_circ_0045997 reverse	CAGACCAGCAGTCAGAGCGT

## Data Availability

The datasets supporting the conclusions of this article are included within the article.

## Abbreviations

CCK-8: Cell Counting Kit-8
circRNA: Circular RNA
ceRNA: Competing endogenous RNA
ECL: Enhanced chemiluminescence
FBS: Fetal bovine serum
FISH: Fluorescence in situ hybridization
GN: Gangliocytoma
IHC: Immunohistochemistry
miRNAs: MicroRNAs
miR-195: MicroRNA-195
MREs: miRNA reaction elements
Mut: Mutant
NC: Negative control
NB: Neuroblastoma
OD: Optical density
PAK2: P21-activated kinase 2
RT-PCR: Quantitative real-time PCR
TEM: Transmission electron microscopy
WT: Wild type.

## Additional Points

*Clinical Perspectives*. (1) Neuroblastoma (NB) is a common malignant embryonal solid tumor in children. Circular RNAs (circRNAs) are characterized by a stable structure, high abundance, and tissue-specific expression. Recent studies have revealed that circRNAs play an essential role in regulating gene expression by functioning as competitive endogenous RNAs (ceRNAs). (2) Our current research proves that hsa_circ_0013401 can induce NB progression through miR-195 to enhance PAK2 expression. (3) Our experimental verification of an hsa_circ_0013401/miR-195/PAK2 axis in NB lays a foundation for the application of hsa_circ_0013401 and its related regulatory pathways (miR-195/PAK2) in the clinical treatment of NB.
